# Acute kidney injury in critically ill COVID-19 infected patients requiring dialysis: experience from India and Pakistan

**DOI:** 10.1186/s12882-022-02931-3

**Published:** 2022-09-08

**Authors:** Urmila Anandh, Amna Noorin, Syed Khurram Shehzad Kazmi, Sooraj Bannur, Syed Shahkar Ahmed Shah, Mehrin Farooq, Gopikrishna Yedlapati, Waseem Amer, Bonthu Prasad, Indranil Dasgupta

**Affiliations:** 1 Department of Nephrology, Yashoda Hospitals, Secunderabad, India; 2 Department of Nephrology, Peshawar Institute of Cardiology, Peshawar, Pakistan; 3 Department of Medicine, Ghurki Trust Teaching Hospital, Lahore, Pakistan; 4 Department of Nephrology, Yashoda Hospitals, Secunderabad, India; 5 Peshawar Institute of Cardiology, Peshawar, Pakistan; 6 Department of General Medicine, Ghurki Trust Teaching Hospital, Lahore, Pakistan; 7 Department of Pulmonology, Yashoda Hospitals, Secunderabad, India; 8 Department of Medicine, Lahore Medical and Dental College and Ghurki Trust Teaching Hospitals, Lahore, Pakistan; 9 Department of Statistics, Yashoda Hospitals, Secunderabad, India; 10 University Hospital Birmingham NHS Foundation Trust, Birmingham, UK; 11 Warwick Medical School, University of Warwick, Coventry, UK

**Keywords:** COVID-19, Acute kidney injury, Continuous renal replacement therapy, Slow low efficiency dialysis

## Abstract

**Background:**

Acute kidney injury (AKI) was common in the first two waves of the SARS-COV-2 pandemic in critically ill patients. A high percentage of these patients required renal replacement therapy and died in the hospital.

**Methods:**

The present study examines the clinical presentation, laboratory parameters and therapeutic interventions in critically ill patients with AKI admitted to the ICU in two centres, one each in India and Pakistan. Patient and outcome details of all critically ill COVID 19 patients admitted to the ICU requiring renal replacement therapy were collected. Data was analysed to detect patient variables associated with mortality.

**Results:**

A total of 1,714 critically ill patients were admitted to the ICUs of the two centres. Of these 393 (22.9%) had severe acute kidney injury (AKIN stage 3) requiring dialysis. Of them, 60.5% were men and the mean (± SD) age was 58.78 (± 14.4) years. At the time of initiation of dialysis, 346 patients (88%) were oligo-anuric. The most frequent dialysis modality in these patients was intermittent hemodialysis (48.1%) followed by slow low efficiency dialysis (44.5%). Two hundred and six (52.4%) patients died. The mortality was higher among the Indian cohort (68.1%) than the Pakistani cohort (43.4%). Older age (age > 50 years), low serum albumin altered sensorium, need for slower forms of renal replacement therapy and ventilatory support were independently associated with mortality.

**Conclusion:**

There was a very high mortality in patients with COVID-19 associated AKI undergoing RRT in the ICUs in this cohort from the Indian sub-continent.

## Background

Since the first description of the severe acute respiratory syndrome (SARS) following a new coronavirus in 2019, [1] the world has been grappling with a pandemic impacting more than 220 countries and territories worldwide. The virus, designated as COVID-19 by WHO, has as of now, affected more than 426 million people with more than 5.9 million dying of this infection. The Indian subcontinent has also seen the devastating impact of this virus.

The kidney is affected in many ways in COVID-19 infection with the most important clinical manifestation being acute kidney injury (AKI) [2–4]. The underlying pathophysiology of AKI is acute tubular necrosis (ATN) which develops secondary to both ischemic (volume depletion, relative intravascular volume deficit in cytokine storm) and nephrotoxic (therapy of COVID-19 infection) insults [5]. There are also some reports of direct cytopathic effect of the virus on the kidney [6]. The incidence of AKI is high in the hospitalized patients as many of the underlying causative factors are present in the critically ill [3]. Almost 20% of critically ill COVID 19 infected patients admitted in the intensive care unit (ICU) need renal replacement therapy (RRT) [7]. The mortality in hospitalised patients with COVID -19 and AKI is relatively high and development of AKI is a poor prognostic factor impacting outcome in patients with COVID -19 pneumonia [3].

In this study, we have examined the clinical presentation, laboratory parameters and therapeutic interventions in critically ill patients with AKI admitted to the ICU in two centres, one each in India and Pakistan, with a view to looking at the prognostic factors for mortality in the Indian sub-continental population.

## Methods

### Study design and patients

A retrospective analyses of the demographic variables, comorbidities, clinical and laboratory parameters, prognostic indicators and outcome data was done of critically ill COVID-19 infected patients requiring dialysis admitted to two ICUs, one in Secunderabad in India and the other in Peshawar, Pakistan. The diagnosis of COVID -19 infection was made when a patient tested positive on a rt-PCR test. Patients with history of underlying chronic kidney disease (CKD) and past laboratory investigations suggestive of CKD were excluded. Data were collected from all consecutive admissions to the two ICUs between April 2020 to December 2020.The subgroup of patients who developed AKI and required renal replacement therapy (RRT) were included for the final analysis.

### Data collection

Demographic data collected included, age, gender and comorbidities (presence of hypertension and diabetes). The clinical presentation of these patients was also studied. Laboratory parameters included routine haematological and chemical parameters, as well as parameters specific to COVID 19 infection(CRP, LDH, Ferritin,Interleukin-6). IL-6 levels were done in a very few patients and as such this was not included in the final analysis. Echocardiographic studies looking at regional wall motion abnormality (RWMA) and left ventricular ejection fraction (LVEF) were performed in all patients. Data on whether the patient was offered intermittent hemodialysis (HD), slow low efficiency dialysis (SLED) or continuous renal replacement therapy (CRRT) was also collected. The type of ventilatory support (invasive or non -invasive) was also noted.

### Statistical analysis

The patient data were collected from Yashoda Hospitals, Secunderabad, India and Peshawar Institute of Cardiology Peshawar, Pakistan. During the pandemic, a unique dashboard was created for all COVID-19 infected patients. Data acquisition, collation and initial analysis of the Pakistani cohort was done with the support of Ghurki Trust Teaching Hospital, Lahore, Pakistan. The demographic, clinical,laboratory parameters and therapeutic interventions were included in the final analysis to look for prognostic factors associated with poor outcome. The final analysis of the data of both the cohorts was done in Yashoda Hospitals, Secunderabad, India.

A Kolmogorov–Smirnov normality test was done and wherever the data followed normal distribution. All continuous variables were depicted as mean (± SD) for normally distributed data. Data not following a normal distribution were represented as mean and inter-quartile range (IQR). Age of the cohort (both India and Pakistan) followed a normal distribution. Hemoglobin and serum albumin levels of the Indian cohort were normally distributed. Rest of the datasets did not follow a normal distribution. Mann Whitney U Test was used to compare whether there were significant differences in the variables studied in the two datasets from India and Pakistan.

A step wise logistic regression analysis was performed to assess the association between the independent variables noted in the study and mortality. The following parameters such as age, comorbidities (diabetes and hypertension), clinical presentation (fever, diarrhea, breathlessness, neurological status at presentation), laboratory parameters (creatinine at admission, creatinine at transfer to ICU, hemoglobin, total leucocyte counts, percentage lymphocyte in the differential count, platelet count, lactate dehydrogenase levels (LDH), c-reactive protein (CRP), ferritin, serum albumin, LVEF, RWMA and the type of respiratory support offered (invasive /noninvasive ventilation), and the type of renal replacement (RRT) offered were included as independent variables.

To test how the model performed, an omnibus test of model coefficients was used. In the model all the independent variables were used. A significance value of 0.000 (< 0.05) was noted which proved that the model was a good fit.The model explained 54.9% (Cox & Snell R Square) and 83.5% (Nagelkerke R Square) of the variance and classified 95.7% cases.

All statistical analyses were done with SPSS version 23 (SPSS Chicago, IL. USA).

## Results

### Demographic and clinical data

During the first wave of the pandemic (between 1^st^ April 2020 to 31^st^ December 2020) a total of 1714 critically ill patients were admitted to the two ICUs. Of these 393 (22.9%) had severe AKI (AKIN Stage 3) requiring RRT. The mean age of this cohort was 58.78 ± 14.4 years and 60.6% (*n* = 238) were males. Seventy-one (49.3%) patients in the Indian cohort and 132 (53.0%) patients in the Pakistani cohort were diabetic (Table 1). Two hundred and seventy-seven patients (70.4%) presented with fever and 350 (89.1%) with breathlessness. Diarrhoea (*n* = 118, 30.0%) and altered sensorium (*n* = 178,45.3%,) were less common clinical presentations. The difference in the clinical presentation between the two centres is shown in Table 1. At the time of initiation of RRT 346 (88.04%) patients were oligo-anuric. The ICU stay ranged from 1 to 60 days (Median 10 days).

**Table 1:** Demography and clinical presentation of the two cohorts of dialysis requiring AKI

Parameter	Combined cohort	Indian Cohort (*n* = 144)	Pakistani Cohort (*n* = 249)	*P* value
Age (Years) Mean ± SD	58.78 ± 14.4	58.48 ± 15.05	58.94 ± 14.05	0.761
Gender Distribution (M:F)	238:155	107:37	131:118	**0.0001**
Presence of Type 2 Diabetes Mellitus (%)	203 (51.6%)	71 (49.3%)	132 (53.0%)	0.479
Fever	277 (70.4%)	74 (51.4%)	203(81.5%)	**0.00001**
Breathlessness	350 (89.1%)	106 (73.6%)	244 (97.9%)	**0.00001**
Diarrhoea	118 (30.0%)	10 (6.9%)	108 (43.4%)	**0.00001**
Altered Sensorium	178 (45.3%)	35 (24.3%)	143 (57.4%)	**0.00001**

### Laboratory data

The laboratory parameters of the cohort (*n* = 393) are given in Table 2. The difference in the initial creatinine in the two cohorts reflect the admission and internal transfer policies. In the Pakistani centre, patients are admitted to a high dependency unit and then transferred to the ICU if dialysis is required. On the other hand, all sick patients are directly admitted to the ICU in the Indian centre. While the Indian cohort had a higher serum creatinine level than the Pakistani cohort at entry to the ICU, patients in Pakistan had higher leucocyte count, CRP, LDH and ferritin values. The serum albumin levels were lower in the Pakistani cohort. Echocardiography was done in 371 patients (94.4% of the cohort); 225 (60.6%) had evidence of RWMA. The LVEF ranged from 21 to 66%.

**Table 2:** Laboratory parameters (Median and IQR) of the cohort

Parameter	Total (*n* = 393)	Indian Cohort (*n* = 144)	Pakistani Cohort (*n* = 249)	Significance
Initial Creatinine (mg/dl)	Median-1.5 IQR -2.9	Median -4.7 IQR—6.0	Median -1.1 IQR -1.0	**0.0001**
Hemoglobin (g/dl)	Median -10.7 IQR -3	Median – 10.85 IQR—4.13	Median—9.0 IQR -3.0	0.257
Total Leucocyte Count (/µL)	Median –20938.3 IQR—14000	Median – 11015 IQR—14000	Median – 23000 IQR—12000	**0.0001**
Percentage Lymphocytes	Median – 9 IQR -16	Median -8.0 IQR -11.05	Median—9.0 IQR -17.0	**0.044**
Platelets (1000/µL)	Median – 200 IQR—145	Median – 200 IQR—100	Median—230 IQR—177	**0.020**
Albumin (g/dl)	Median 3 IQR 1.1	Median -3.4 IQR -0.93	Median -2.9 IQR -1.2	**0.0001**
LDH (IU/ml)	Median -980 IQR -462	Median -482 IQR -345	Median -1000 IQR -244	**0.0001**
CRP (ng/dl)	Median – 13 IQR – 13.9	Median – 6 IQR – 13.9	Median – 16 IQR—13.3	**0.0001**
Ferritin (ng/ml)	Median -1100 IQR—321.3	Median -545 IQR -972	Median -1200 IQR -278	**0.0001**
Ejection Fraction (%)	Median -58 IQR—17	Median -60 IQR -6	Median – 50 IQR -10	**0.001**

### Therapeutic interventions ( RRT and ventilation)

All patients were on therapy for COVID-19 infection as dictated by the policies of the respective governments. This included anticoagulants, remdesivir, tocilizumab and corticosteroids. Antibiotics were offered to all critically ill patients. Antibiotic use was dictated by the respective intensive care unit. One hundred and ninety-four (49.4%) patients needed non-invasive ventilation and another 92 (23.4%) were ventilated invasively. The rest (*n* = 107) required nasal oxygen supplementation.106 patients (44 in the Pakistan cohort and 62 in the Indian cohort) were on ionotropic support. Intermittent HD was offered to 183 (46.5%, Pakistan 132, India 51), SLED was offered to 175 ( 44.5%, 117 in Pakistan, 58 in India) patients. CRRT was offered to 35 (8.9%) patients in India. CRRT was not offered to any patient in Pakistan. The modality of RRT was determined by the underlying clinical condition of the patient and the available resources. No patient was offered peritoneal dialysis.

### Outcome

Two hundred and six patients (52.4%) died during their intensive care stay; 97.8% of the patients who were on invasive ventilation died.The percentage mortality in the Indian cohort (68.1%) was higher than the Pakistani cohort (43.4%).

### Factors influencing outcome

The stepwise regression analysis data are presented in Table 3 which shows that age (>50 years), presentation with altered sensorium, low serum albumin, requirement of ventilatory support (non-invasive/ invasive ventilation) and requirement of SLED/CRRT were independently associated with mortality. (*p* < 0.05). A low initial serum creatinine at presentation and absence of RWMA on echocardiography were associated with lower mortality. Diabetes and CRP were not associated with mortality

**Table 3:** Stepwise logistic regression showing factors predicting mortality

Parameter	OR	95% CI	*p*-value
Age > 50 years	3.501	1.696–7.224	**.001**
Low Serum Creatinine at admission	.692	0.593–0.808	.**000**
Low Serum Albumin	2.389	1.478—3.861	**.000**
No RWMA	0.357	0.150–0.853	.**020**
Neurological Status	5.380	2.570 -11.259	**.000**
Type of Dialysis (SLED/CRRT)	9.354	4.076 – 21.465	**.000**
Ventilation (Invasive/Non Invasive)	5.205	1.645–16.464	**.005**

## Discussion

In this study of 1,714 consecutive patients with COVID-19 infection admitted to two ICUs in India and Pakistan, during the first wave of the pandemic, there was a high incidence of AKI requiring renal replacement therapy (22.9%). The mortality rate among the COVID-19 AKI patients requiring renal replacement therapy was 52.4%. Ninety eight percent of patients who required invasive ventilation died in hospital. The mortality was higher among the Indian cohort (68.1%) than the Pakistani cohort (43.4%). Whilst older age, presentation with altered sensorium, requirement of SLED/CRRT and ventilation (non-invasive or invasive) were independent predictors of mortality; lower serum creatinine at presentation and absence of regional wall motion abnormalities on echocardiography were good prognostic factors.

The incidence of AKI secondary to COVID-19 depends on the clinical setting and the parameters used to diagnose AKI. Most studies have reported an incidence of 30–50% in hospitalised patients [3, 8, 9]. The mortality in hospitalised patients with COVID -19 and AKI is relatively high and the development of AKI is a poor prognostic factor impacting outcome in patients with COVID -19 pneumonia [10, 11]. About 30–40% of COVID-19 infected patients in the ICU require RRT [11, 12]. The mortality is higher in critically ill patients admitted to the ICU. In the first part of the pandemic in 2020 it was around 45% [13].

AKI requiring RRT in patients with COVID-19 pneumonia reflect a severe form of illness. Patients who develop AKI are usually older, have high initial Sepsis-Related Organ Failure Assessment (SOFA) scores, have multi-organ dysfunction and often need invasive ventilation [14, 15]. In this group of patients, around 20% of patients require renal replacement therapy (AKI-RRT) [12]. The incidence in our cohort from the Indian subcontinent is similar, with 22.9% of critically ill patients needing RRT.

AKI -RRT subgroup of patients represent the most severe form of AKI to intensivists as RRT in critically ill patients is often a challenge [5]. Most of these patients have hemodynamic instability and are better managed with CRRT. However, the severe crunch of dialytic resources during the pandemic led to various improvisations in the delivery of dialytic therapy. In the developed world, most patients received CRRT [16]; however, in resource constrained countries prolonged intermittent renal replacement therapy (PIRRT) and slow low efficiency dialysis (SLEDD) were the mainstays of RRT [17]. In our cohorts, even though many would have ideally qualified for CRRT, majority were offered either SLED (44.5%) or intermittent hemodialysis (46.5%). In the Pakistani cohort, the most common dialytic modality was intermittent hemodialysis (53.0%); none was offered CRRT.

Mortality in AKI-RRT subgroup is reported to be as high as 60–80% in many studies [7, 16]. The overall mortality (52.4%) in our subset is similar to many studies reported in the literature [18–20]. Many factors have been identified to be associated with poor outcome. Severity of AKI is a consistent prognostic indicator. Other factors which have shown to be important in multivariable analysis are older age group, presence of diabetes, underlying CKD and the number of ICU beds [21–23]. In our study, age over 50 years was associated with mortality but diabetes mellitus was not. We excluded all patients known to have CKD, but were not able to establish how many of our cohort had underlying unrecognised CKD. However, lower serum creatinine at presentation was associated with better outcome.

Requirement of longer-duration dialysis (SLED or CRRT) was associated with a poor outcome, probably reflecting the overall unstable hemodynamic status of these patients. Similarly, patients who were ventilated (either non-invasively or invasively) also had a poorer outcome. These findings are consistent with observations from other parts of the world [24–28].

There are a number of limitations to this study, the first being it is retrospective in nature. During the pandemic the resources available to the medical professionals were stretched to the limits in the developing world making it difficult to conduct a prospective study. Secondly, we did not collect the AKI recovery data which was beyond the purview of this analysis. Finally, the data were from two different countries with disparate clinical practices, hence generalisation may not be appropriate. However, this study portrays the status of management of COVID-19 AKI in a subcontinental cohort during the first wave of the pandemic. The results may help to guide physicians in resource-poor settings to manage COVID 19 AKI-RRT patients in the future.

Furthermore, the wide range of information collected in a large cohort of patients on clinical, laboratory variables and therapeutic intervention parameters from two different countries make our study an important document on prognostication in COVID -19 patients admitted in ICU requiring RRT in the Indian sub-continent. This is important as the care of this group of patients is extremely resource intensive, and the outcome is very grim [29, 30]. A very high mortality in a subgroup of patients requiring invasive ventilation and RRT may temper our clinical decision whether to escalate therapy in these unfortunate patients in resource constrained settings. To the best of our knowledge, this is the first study describing AKI outcomes in critically ill COVID -19 patients admitted to ICU requiring RRT from the Indian subcontinent.

## Conclusions

AKI requiring RRT in critically ill COVID-19 pneumonia patients in this sub-continental cohort had a high mortality which was independently predicted by age, altered sensorium at presentation, need for ventilatory support and RRT. We hope the results of this study will help to prognosticate critically-ill COVID-19 AKI patients and direct therapy appropriately in resource constrained settings.

## Data Availability

All data and materials are available with the corresponding author and will be made available on reasonable request.
